# Increased Recovery of Gold Thiosulfate Alkaline Solutions by Adding Thiol Groups in the Porous Structure of Activated Carbon

**DOI:** 10.3390/molecules25122902

**Published:** 2020-06-24

**Authors:** Freddy R. Escobar-Ledesma, Carlos F. Aragón-Tobar, Patricio J. Espinoza-Montero, Ernesto de la Torre-Chauvin

**Affiliations:** 1 Department of Extractive Metallurgy, Escuela Politécnica Nacional, Ladrón de Guevara E11-253, P.O. Box 17-01-2759, Quito 170525, Ecuador; carlos.aragont@epn.edu.ec; 2 Escuela de Ciencias Químicas, Pontificia Universidad Católica del Ecuador, Avenida 12 de Octubre y Roca, Apartado: 17-01-2184, Quito 170525, Pichincha, Ecuador; pespinoza646@puce.edu.ec

**Keywords:** activated carbon, adsorption, gold thiosulphate complex, gold thioglycolate complex

## Abstract

Thiosulfate leaching combined with ion-exchange resins is an innovative alternative for gold recovery. According to the properties of activated carbon, it could replace resins in the gold recovery process, improve efficiency, and reduce operating cost. In this research, the adsorption process of gold thiosulfate complex on thiol-modified activated carbon was studied. Thioglycolic acid (ATG) was impregnated in activated carbon, and its adsorption ability was tested with synthetic solutions of gold and sodium thiosulfate (Au 10 mg·L^−1^, Na_2_S_2_O_3_ 0.1 mol·L^−1^, pH = 10.0). Carbon was characterized by infrared spectroscopy, SEM-EDS, PZC titration, hardness number measures, and proximal analysis. Synthetic solutions were also characterized by UV-vis spectroscopy and cyclic voltammetry. The percentage of volatile material increased from 10.0 to 13.9% due to the impregnation process of ATG. Infrared spectra show characteristic bands of C-H, S-H, and C-S bonds. In the adsorption tests, the ATG-impregnated carbon achieved 91% of gold recovery, while the same amount of ATG in the liquid phase stirred with unmodified activated carbon reached 90% of gold recovery. The 44.9% of gold recovered with activated carbon impregnated with ATG was eluted with sodium cyanide ([NaCN] = 0.2 mol·L^−1^; [NaOH] = 0.25 mol·L^−1^; [CH_3_CH_2_OH] = 30% *V*/*V*; pH = 12.0; t = 24 h). These results suggest the gold transferred from the thiosulfate complex to a new gold thiolate complex.

## 1. Introduction

Cyanidation has been the most used technique for the hydrometallurgical gold recovery. It is simple, cheap, and effective, consequently, it has been positioned as the best alternative for gold-ores processing [1,2]. However, cyanide is also dangerous for the environment due to its high toxicity, which has motivated researchers to seek new alternatives for gold processing. Aqua regia, chlorine gas, hypochlorite, thiourea, dithiooxamide, and thiosulfate have been studied as replacements for cyanide salts [2,3,4]. However, as an alternative to cyanidation in gold recovery, thiosulfate leaching has been reordered. Thiosulfate leaching appears to be an innovative and eco-friendly alternative for the upcoming replacement of cyanidation; besides, thiosulfate is cheaper than cyanide. In addition, thiosulfate leaching is effective for copper and arsenic ores and does not get polluted with undesirable metal ions, the opposite of what happens with cyanide effluents [2,5].

Gold can be dissolved at room temperature with oxygen and thiosulfate as a ligand, but the reaction is slower. Copper and ammonia improve the gold dissolution and increase the reaction rate by 20 times, according to the following reactions [2]. In this case, ammonia and copper are combined to form a cupric tetra-amine complex, which is a strong oxidizing agent that improves the gold leaching [4,5].

(1) $${\mathrm{Au}}^{+}+2{\mathrm{NH}}_{3}⇄\mathrm{Au}{\left({\mathrm{NH}}_{3}\right)}_{2}{}^{+}\;\Delta G{}^{\circ}=-74.1\;\mathrm{kJ}\times {\mathrm{mol}}^{-1}$$

(2) $$\mathrm{Au}{\left({\mathrm{NH}}_{3}\right)}_{2}{}^{+}+2S_{2}O_{3}{}^{2-}⇄2{\mathrm{NH}}_{3}+\mathrm{Au}{\left(S_{2}O_{3}\right)}_{2}{}^{3-}\;\Delta G{}^{\circ}=-74.9\;\mathrm{kJ}\times {\mathrm{mol}}^{-1}$$

(3) $$\mathrm{Cu}{\left({\mathrm{NH}}_{3}\right)}_{4}{}^{2+}+3S_{2}O_{3}{}^{2-}⇄\;\mathrm{Cu}{\left(S_{2}O_{3}\right)}_{2}{}^{5-}+4{\mathrm{NH}}_{3}\;\Delta G{}^{\circ}=-21.9\;\mathrm{kJ}\times {\mathrm{mol}}^{-1}$$

(4) $$4\mathrm{Cu}{\left(S_{2}O_{3}\right)}_{3}{}^{5-}+16{\mathrm{NH}}_{3}+O_{2}+2H_{2}O⇄4\mathrm{Cu}{\left({\mathrm{NH}}_{3}\right)}_{4}{}^{2+}+4{\mathrm{OH}}^{-}+12S_{2}O_{3}{}^{2-}\;\Delta G{}^{\circ}=-67.1\;\mathrm{kJ}\times {\mathrm{mol}}^{-1}$$

(5) $$2\mathrm{Cu}{\left({\mathrm{NH}}_{3}\right)}_{4}{}^{2+}+8S_{2}O_{3}{}^{2-}⇄\;2\mathrm{Cu}{\left(S_{2}O_{3}\right)}_{3}{}^{5-}+S_{4}O_{6}{}^{2-}+8{\mathrm{NH}}_{3}\;\Delta G{}^{\circ}=-19.8\;\mathrm{kJ}\times {\mathrm{mol}}^{-1}$$

Thiosulfate leaching has been tested for sulfide ores, carbonaceous ores, roasted gold concentrates, and electronic waste [6,7,8]. Cementation, ionic flotation, and ion-exchange resins are often used to recover gold from pregnant solutions. These processes have operational disadvantages since liquors must be separated from the pulp. Besides, the high cost of resins renders these processes expensive [2,3,4,9]. Moreover, activated carbon could be used instead of resins to reduce operative costs and improve efficiency. However, carbon has proven to be ineffective for adsorbing gold from thiosulfate leach solutions because of the high anionic charge and steric limitations of the complex [3,10,11,12]. Nevertheless, the modification of the surface of activated carbon would allow for the recovery of the gold thiosulfate complex with activated carbon, as has been presented in previous studies [13,14]. In fact, organic groups such as thiols or amines can be attached on the surface of activated carbon to improve the gold thiosulfate affinity. Consequently, adsorption with modified activated carbon could be a viable solution.

Sulfur complexes are used to adsorb metal ions, such as cadmium, mercury, or lead due to the formation of strong bonds between metals and carbon-sulfur complexes [13]. Likewise, adsorption of the gold thiosulfate complex has been successfully tested using adsorbent materials modified with sulfur groups, such as silica gel and activated carbon. Thiol and disulfide groups have been attached to the surface of activated carbon by impregnation with MBT (2-mercaptobenzothiazole) in alcohol solutions. This activated carbon recovers 95% of gold from solutions with 10 mg·L^−1^ of the gold thiosulfate complex [15]. Quaternary amines have also been impregnated in carbon nanotubes using the same procedure, for instance, Zhou et al. [16] studied the impregnation of polyethyleneimine in carbon nanotubes previously oxidized with HNO_3_ (5 mol·L^−1^) at room temperature for CO_2_ capture applications [16]. In the same way, silica mesoporous gel was prepared through sol–gel synthesis using mercaptosilanes as precursors in order to study its ability to adsorb gold thiosulfate complex. It achieved gold recoveries higher than 90% after 24 h of stirring in synthetic solutions of 50 mg·L^−1^ of gold [17]. Finally, Chen et al. [14] reported up to 90% of gold recovery by an activated carbon impregnated with 1-phenyl-5-mercaptotetrazole (PMT), and suggested a ligand interchange as a mechanism for gold recovery [14].

Literature shows that adsorption ability of activated carbon mainly depends on its large surface area. However, the groups attached to the carbon surface constitute active centers susceptible to physical or chemical interactions. Thus, this property allows for the incorporation of sulfur functionalities capable of interacting with the gold thiosulfate complex and recovering it from leaching solutions [18,19,20,21]. In this paper, thioglycolic acid (ATG) was impregnated on microporous activated carbon in order to study its gold adsorption ability. Results showed that ATG improved the adsorption capacity and increased the gold recovery when it was impregnated on the carbon. Importantly, the mechanism of gold recovery for this system was also proposed.

## 2. Results and Discussion

### 2.1. Gold Adsorption Studies

#### 2.1.1. Influence of ATG in Solution on Gold Adsorption

**Figure 1:**
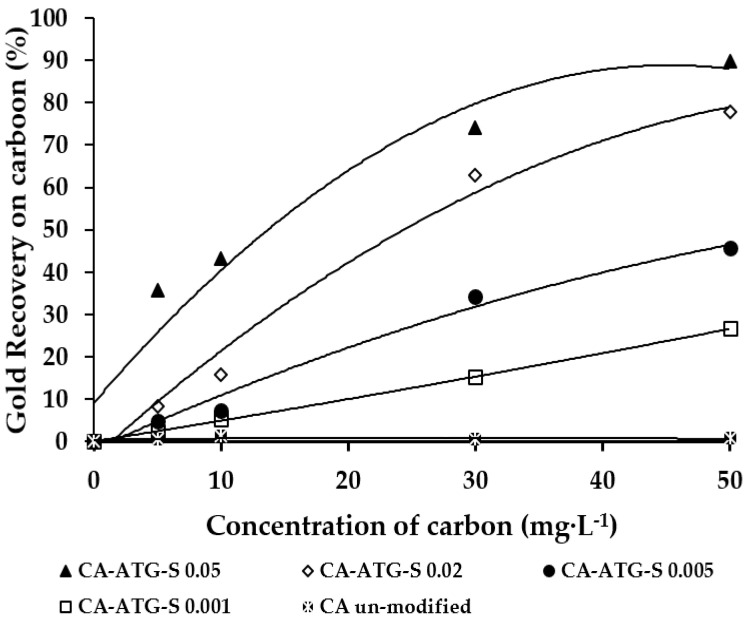
Gold recovery curves in gold thiosulfate synthetic solutions (10 mg·L^−1^ Au and 0.1 mol·L^−1^ Na_2_S_2_O_3_), thioglycolic acid (ATG) stirred in liquid phase (0.001, 0.005, 0.02, and 0.05 mol·L^−1^) and carbon concentrations (5, 10, 30, and 50 g·L^−1^) at room temperature (15 °C).

Gold adsorption with ATG in aqueous solution was studied at four different concentrations of ATG (0.001, 0.005, 0.02, and 0.05 mol·L^−1^) and carbon (5, 10, 30, and 50 g·L^−1^) using 50 mL of synthetic solution (10 mg·L^−1^ Au and 0.1 mol·L^−1^ Na_2_S_2_O_3_) at room temperature (15 °C). Figure 1 presents the gold recovery curves, showing an exponential behavior and a remarkable influence of ATG concentration on the gold recovery. If carbon without ATG was used, the gold recovery was practically 0%, but when the ATG was added to the aqueous solution (0.05 mol·L^−1^ of ATG and 50 g·L^−1^ of carbon) the gold recovery increased to 90%. Gold recovery increased also with the concentration of carbon. However, the equilibrium value was reached with a lower amount of carbon (50 g·L^−1^ of carbon) at higher ATG concentrations (0.05 and 0.02 mol·L^−1^ of ATG). These results agree with a previous study, for instance, when 2-mercaptobenzothiazole (MBT) was impregnated in carbon at different concentrations (0.1, 0.3, 0.5, 0.7, and 0.9 g of MBT) and different contact times (8, 12, 24, 48, and 72 h with 0.7 g MBT), the gold recovery depended directly on the concentration of MBT used in the impregnation procedure [15].

#### 2.1.2. Influence of Activated Carbon Impregnated with ATG on Gold Adsorption

**Figure 2:**
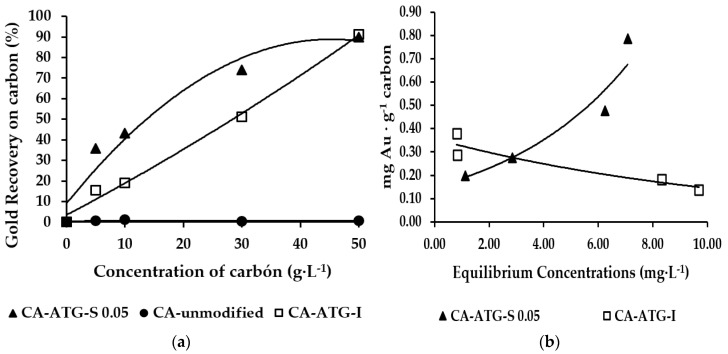
Gold adsorption with ATG-impregnated carbon and ATG stirred in liquid phase 0.05 mol·L^−1^: (**a**) gold recovery curves, (**b**) isotherms (where **S** represents activated carbon stirred with ATG in aqueous solution and **I** represent activated carbon impregnated with ATG).

Figure 2a shows the gold recovery curve in reference to the adsorption assays with activated carbon impregnated with ATG. The adsorption assays were conducted using 50 mL of synthetic solution (10 mg·L^−1^ Au and 0.1 mol·L^−1^ Na_2_S_2_O_3_) at room temperature (15 °C). This curve shows a roughly linear trend in the carbon concentrations tested; however, it is expected that this curve will become asymptotic as the concentration of carbon rises. Figure 2a also shows the best gold recovery curve from Section 2.1.1, which corresponds to an ATG concentration of 0.05 mol·L^−1^. There was no difference in gold adsorption between ATG stirred in aqueous solution and ATG impregnated in carbon. In fact, both activated carbons reached 91% gold recovery, which contrasts with unmodified carbon that does not have ability to recover gold. However, the gold recovery on the activated carbon impregnated with ATG was lower when the amount of carbon decreased (5, 15, and 30 g·L^−1^). This difference in gold recovery is explained by the amount of ATG available to react with gold. On the one hand, when the thioglycolic acid is in an aqueous media, it is immediately accessed to form the gold complex and the gold recovery process is more efficient. On the other hand, when the thioglycolic acid is impregnated on the carbon surface, the gold thiosulfate complex must first reach the surface of the carbon before gold can transfer inside the porous carbon. Thus, a mass transfer resistance is added to the adsorption process [14,22]. The isotherms presented in Figure 2b also evidence this behavior. The adsorption of activated carbon with ATG (0.05 mol·L^−1^) stirred in aqueous solution is better than the adsorption of carbon impregnated with ATG. This improvement observed in the absorption is linked with the equivalent concentration of ATG in the impregnated carbon, which is approximately 0.02 mol·L^−1^.

#### 2.1.3. Influence of pH on the ATG Adsorption Tests

**Figure 3:**
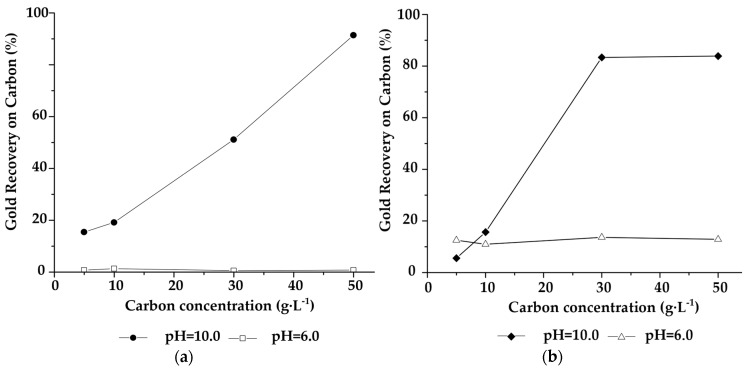
Gold adsorption curves at pH = 10.0 and pH = 6.0 (T = 15 °C), (**a**) activated carbon impregnated with ATG, (**b**) activated carbon stirred with ATG in aqueous solution.

Melashlvili et al. [23] state that thiosulfate is an unstable species that easily disproportions with other metastable species such as tetrathionate (S_4_O_6_^−2^), trionate (S_3_O_6_^−2^), sulphite (SO_3_^−2^), and sulfur (S^−2^). Therefore, leaching with thiosulfate should be completed with a thorough control of the pH to prevent its decomposition and improve the formation of the gold complex. In this sense, adsorption tests were carried out at pH = 10.0 [23,24]. However, to assess the stability of the thiosulfate complex with ATG, gold adsorption tests were also performed at pH 6.0 with ATG-impregnated activated carbon (CA-ATG-I) and unmodified activated carbon stirred with ATG in aqueous solution (CA-ATG-S). Figure 3 shows the gold recovery as a function of the amount of carbon; in both cases, carbons lost the ability to recover gold when the pH was modified from 10.0 to 6.0.

#### 2.1.4. Experiment for Gold Stripping from ATG-Impregnated Carbon

Table 1 presents the gold recoveries when four elution solutions were used. The highest elution percentage corresponded to sodium cyanide with 44.9%. The low gold recoveries in all eluents tested suggest a high affinity of the gold complex adsorbed by carbon, a similar behavior has been reported between the gold-thiosulfate complex and ion-exchange resins [12,25]. They also suggest that gold is transferred to another new complex with a greater affinity for carbon. Chen et al. [14] also proposed a mechanism of exchange of ligands; in Section 2.5, this topic is described in more detail when the proposed mechanism of adsorption is presented [14]. Here, it is important to emphasize that the gold mining industry consumes large amounts of activated carbon, despite the fact that it is regenerated and reused as much as technologically possible. The gold mining industry will accept new technology as long as the recovery of gold from loaded carbon is possible. In this sense, it is vital to suggest a compound with sufficient ability to strip gold from activated carbon. The cyanidation-CIP (CIP: carbon in pulp) process uses cyanide in two unit operations: gold leaching and gold elution. Although the usage of cyanide is considered an environmental problem, gold leaching is where most cyanide is used. Water used in gold elution is approximately 10% of the volume in cyanidation, and it is recycled up to 100%. Likewise, it is important to remark that using thiosulfate instead of cyanide in the gold leaching step would constitute an advancement towards displacing cyanidation as the main technology to process gold ores [1,26,27].

### 2.2. Specific Surface, Volatile Matter Content, and Zero Charge pH of Activated Carbons

**Figure A1:**
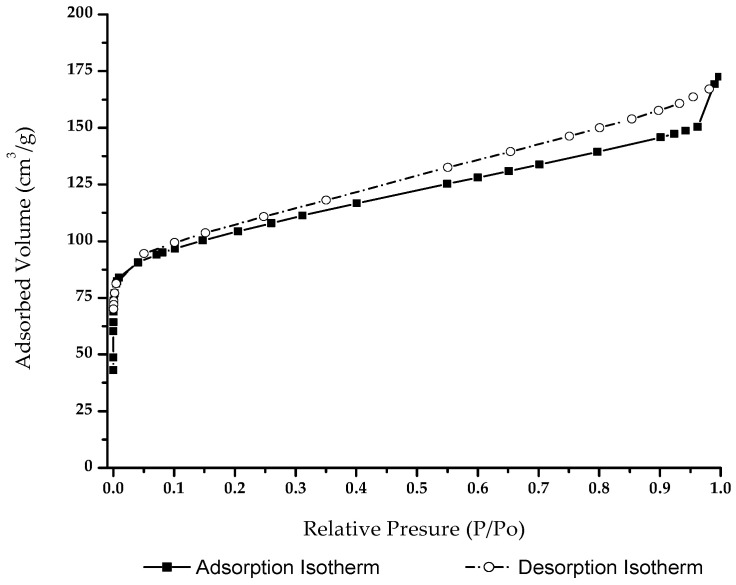
N_2_ adsorption–desorption isotherms of unmodified carbon.

**Figure A2:**
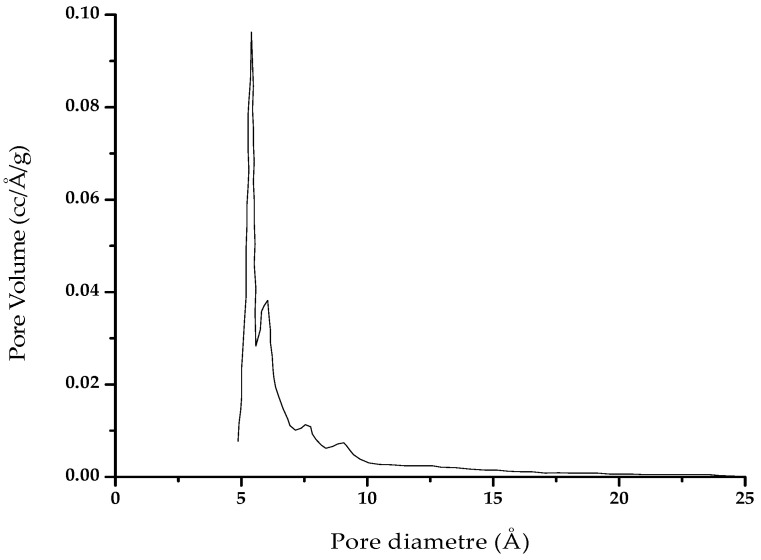
Pore distribution of unmodified carbon, Horvath–Kawazoe model.

The properties of unmodified carbon and carbon impregnated with ATG are compared in Table 2. The volatile material content increased from 10.0 to 13.9% as a consequence of the impregnation process. In the same way as a result of the impregnation process, the hardness remained around 90.0% and the point of zero charge reduced from 9.8 to 3.2. The increase in volatile material and the decrease in the point of zero charge (PZC) confirmed the impregnation of ATG on carbon since the basic groups on the carbon surface were neutralized [28]. The values of zero charge pH (PZC) and the total acidity of the unmodified carbon reveal the presence of functional groups such as quinones, carbonyls, or ethers. However, the basicity of carbon is also associated with the π bonds of the aromatic rings of graphene [20]. The specific surface area of unmodified carbon was 274 m^2^ × g^−1^. N_2_ adsorption isotherms of unmodified carbon and pore distribution are displayed in Appendix A (Figure A1 and Figure A2). As it is presented in Figure A1, adsorption–desorption isotherms of unmodified carbon present hysteresis type H4, which is normally observed in microporous adsorbents. The pore diameter of carbons activated by water-steam and CO_2_ is between 0.5 and 1.5 nm. Since the size of the molecule of the gold thiosulfate complex is 0.6 nm, approximately, it is determined that the anionic charge of the complex is the predominant factor of low affinity of the complex for carbon [29].

**Figure 4:**
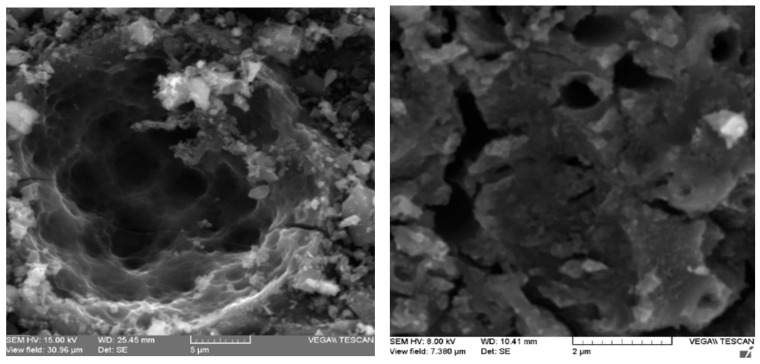
Scanning electron microscopy (SEM) images of thiol-impregnated activated carbon.

**Figure A3:**
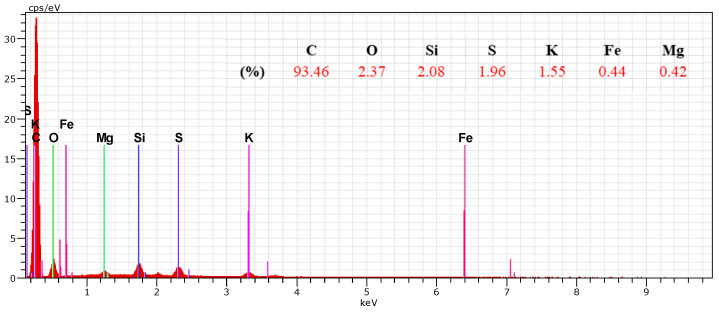
Elemental analysis of the thiol-impregnated activated carbon.

Additionally, the structure, morphology, and elemental analysis of the thiol-impregnated activated carbon were performed with a scanning electron microscope with X-ray dispersion microanalyzer (SEM-EDS). The SEM images in Figure 4 show continuous micropores and mesopores, which should play an important role in ATG impregnation, promoting gold transfer. The elemental analysis presented in Figure A3 (Appendix B) shows that the most abundant element is carbon (93%), followed by oxygen (2.3%), silicon (2.0%), and sulfur (1.9%). The sulfur content is remarkable since it is in accordance with the increase of volatile matter content, and it is directly related to the impregnation of thioglycolic acid. On the other hand, gold adsorbed is not detected by this method; thereby the determination of the gold content was carried out by fire assay and atomic absorption spectroscopy. The gold balance of a sample of carbon obtained from an adsorption test is presented in Table A2 (Appendix B). This gold balance evidences a gold recovery of 89% in the activated carbon.

### 2.3. FTIR Characterization of Activated Carbon Impregnated with ATG

**Figure 5:**
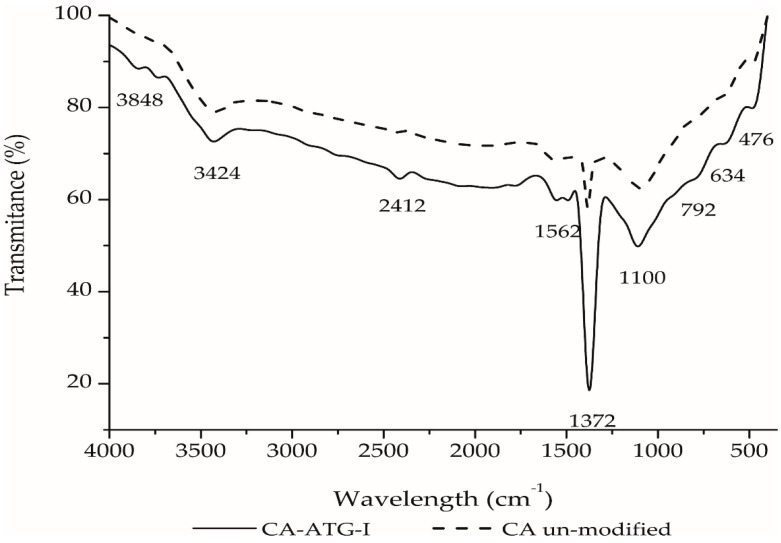
FTIR spectra of activated carbons: activated carbon impregnated with ATG (CA-ATG-I) and original carbon (CA unmodified).

A comparison of the infrared spectra of unmodified carbon and carbon impregnated with ATG are shown in Figure 5. A weak band appears at approximately 2410 cm^−1^, as evidence of sulfur functionalization. In fact, the stretching of the SH bond is typically shown in the region between 2590 and 2450 cm^−1^. Likewise, the activity of the C-S link is seen between 480 and 630 cm^−1^ [14,17]. Other typical bands of activated carbon are observed at 1100 and 1562 cm^−1^, which are assigned to strength vibrations of C-O and C=O bonds. Likewise, a band appears at 3424 cm^−1^, which is attributed to strength vibrations of the hydroxyl group [20,30].

### 2.4. Cyclic Voltammetry of Gold Thiosulphate and Gold Thiolate Synthetic Solutions

**Figure 6:**
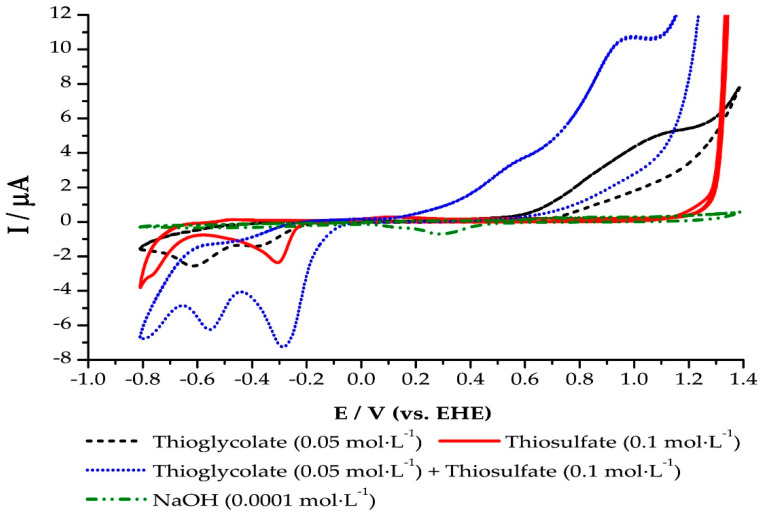
Cyclic voltammogram of gold thiosulfate and thioglycolate synthetic solutions.

The voltammograms presented in Figure 6 were obtained from the Au/thiosulfate 0.1 mol·L^−1^ and Au/Thioglycolate 0.05 mol·L^−1^ systems with a scanning speed of 100 mV × s^−1^ and gold as a working electrode. In both systems, a solution of NaOH 1 × 10^−4^ mol·L^−1^ was used as a supporting electrolyte. During the direct sweep from 1.20 to −1.00 V, in the cathodic direction, it was possible to observe the formation of several peaks corresponding to the gold reduction at −0.60, −0.55, and −0.30 V. Peaks of oxidation were also observed at 1.0 and 1.2 V in the reverse scan. The voltammogram of supporting electrolyte is also presented in Figure 6. No peaks were observed during the forward and reverse exploration of the supporting electrolyte, and, therefore, the presence of two different gold complexes was confirmed [31,32]. Finally, the voltammogram corresponding to the Au/(Thiosulfate 0.10 mol·L^−1^ + thioglycolate 0.05 mol·L^−1^) system is also presented, it is shown as a combination of voltammograms of the Au/thiosulfate and Au/Thioglycolate systems.

### 2.5. Adsorption Mechanism

**Figure 7:**
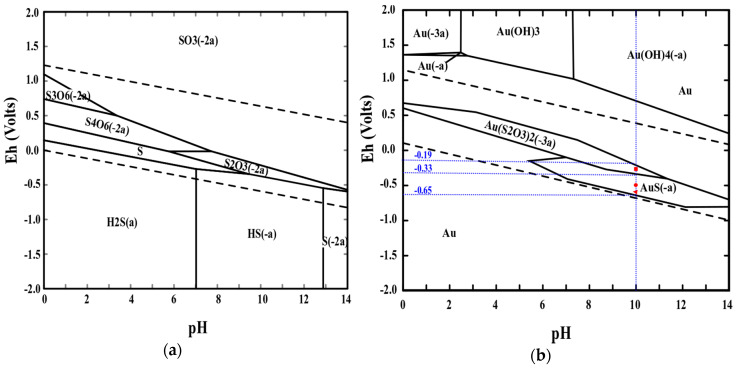
Eh-pH diagrams: (**a**) S-H_2_O system and (**b**) Au-S-H_2_O system.

The high gold recovery observed in carbons modified with ATG is explained from the Eh-pH diagrams for the S-H_2_O and Au-S-H_2_O systems, which are shown in Figure 7a,b, respectively. Thiosulfate, (S_2_O_3_)^−2^, sulfide, S^−2^, and hydrogen sulfide, HS^−^, are stable from pH 6.0 to 14.0, hence, several gold complexes could coexist in this system. Gold as sulfide, AuS, predominates at pH values higher than 12, while gold thiosulfate complex, (Au(S_2_O_3_)_2_)^−3^, is stable between pH 6.0 and 12.0 [23,24,33]. At pH 10.0, ATG can interact with gold to form a complex with more affinity for activated carbon than that of the gold thiosulfate complex. Gold recovery decreased from 91% to 11% when the value of pH passed from 10.0 to 6.0. It can be inferred that gold recovery is directly related to thiol groups because at pH 6 the thiolate complex does not form [14].

The affinity of organic thiol compounds for gold was proven twenty years ago. Researches in electronics technology have used this property to coat gold plates and study their applications. Besides, thiols in excess combined with a reducing agent are used to obtain gold nanoparticles [32,34,35]. Similarly, this affinity is also evident in a typical leaching operation, in which sulfur from sulfide ores minerals is adsorbed on the surface of gold, causing a detrimental effect on the gold recovery because of the surface passivation [1,33,36].

As mentioned in Section 2.4, several peaks appeared on cyclic voltammograms (Figure 6), they were attributed to gold thiolate and gold thiosulfate complexes. Each reduction potential was then compared to the stability region of each complex according to the Eh-pH diagram presented in Figure 7b at pH 10.0, which was the pH of the electrochemical tests. The peak at −0.30 V corresponds to the gold thiosulfate complex and these complexes are stable between −0.19 and −0.33 V. Peaks at −0.6 and −0.55 V are located within the stable region of the gold thiolate complexes (between −0.33 and −0.65 V). These two peaks could correspond to gold thioglycolate and gold sulfide, respectively. Finally, peaks that appear in the anodic region could be attributed to sodium thiosulfate and sodium hydrogen sulfite species [14,23,24].

**Figure 8:**
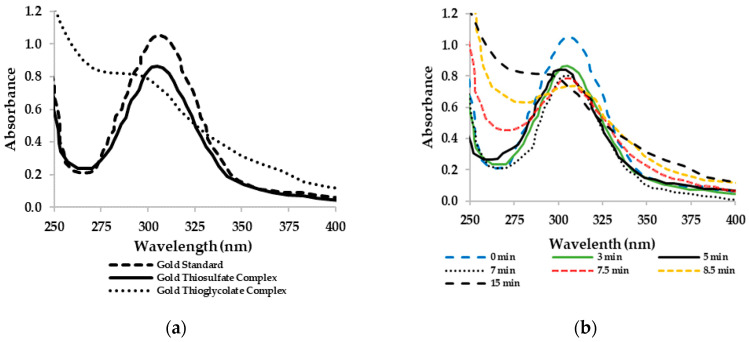
UV-VIS spectra of gold complex solutions: (**a**) chloroauric standard, gold thiosulfate, and gold thioglycolate; (**b**) gold thioglycolate evolution as function of time.

The interaction between the thiol groups and the gold ions is also noticed on the UV-visible spectra of gold complex solutions, which are displayed in Figure 8. Figure 8a compares the UV-visible spectrum of gold standard (50 mg·L^−1^) with two spectra for gold-thiol complexes. The first one is the UV-visible spectra of a mixture of gold standard (50 mg·L^−1^) with sodium thiosulfate (0.01 mol·L^−1^), whereas the second one corresponds to a mixture of a gold standard (50 mg·L^−1^) with thioglycolate (0.05 mol·L^−1^). Thanks to these spectra, is it possible to identify the appearance of several species. Thus, at 312 nm two bands of maximum absorption appear, the first one corresponds to the chloroauric complex while the lower one is a consequence of the gold transfer between chloroauric and thiosulfate complexes. On the other hand, the interaction between gold ions and thioglycolate is more evident due to the displacement of the absorption band from 312 to 304 nm. Additionally, Figure 8b shows the evolution in the UV-visible spectra as a consequence of adding thioglycolate to a solution of gold standard (50 mg·L^−1^). This behavior supports the theory that the thioglycolate and thiosulfate molecules interact in competitive equilibrium and agree with the results obtained by cyclic voltammetry. The same behavior was observed in the spectra of gold complexes formed with guanidine, pyridine and tetracyanide-l-cysteine derivatives and gold pyridine [37,38,39].

The adsorption of gold in activated carbon is clearly related to the presence of thioglycolic acid. The thiol groups, especially sodium thioglycolate, can reduce disulfide bonds, later, these can be re-oxidized by insertion of transition metal ions. This property is used to obtain nanoparticles of keratin metal complexes [40]. In this sense, the thiol group of the thioglycolate anion could break the disulfide bond (S-S) of the gold thiosulfate complex. Thus, two intermediate species could be formed and rearranged to form a gold thioglycolate complex and sodium hydrogen sulfite [41,42,43].

Similar to this research, Chen et al. [14] proposed two routes to form the final product (RS-Au-SR) after analyzing the binding energies of activated carbon impregnated with phenyl-5-mercaptotetrazole (PMT) by X-ray photoelectron spectroscopy (XPS). The insertion of sulfur groups onto carbon in four markedly different chemical environments were demonstrated by the XPS results. These were thiol (R-S^−^), disulfide (R-S-S-R), sulfinic acid (R-SO_2_^−^), and sulfonic acid (R-SO_3_^−^). Subsequently, as a result of gold adsorption, the binding energies of the thiol and disulfide groups increased, which means that only these two groups give modified carbon the ability to recover gold from thiosulfate solutions [14,41].

**Figure 9:**
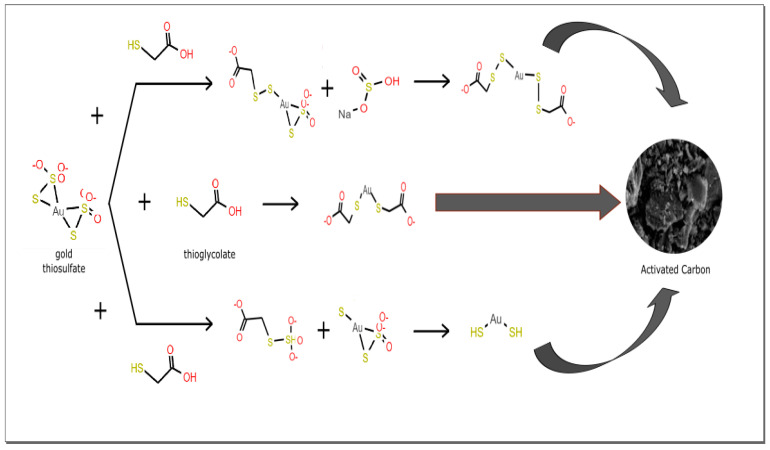
Proposed mechanism of gold recovery with unmodified activated carbon stirred with ATG in aqueous solution.

These statements agree with adsorption experiments, electrochemical measurements, and spectroscopic characterization of the complexes in aqueous solution, and suggest that the adsorption process may involve a ligand exchange between thiosulfate and thioglycolate, according to Figure 9 [14].

## 3. Materials and Methods

### 3.1. Materials

The following analytical grade reagents were used: standard gold solution (Accusatandart, 1000 mg·L^−1^ Au, 5% HCl); anhydrous sodium thiosulfate Na_2_(S_2_O_3_)^−2^ (Panreac, 99%); thioglycolic acid ATG (Merck, 80%); sodium hydroxide NaOH (Panareac, 99%); sodium nitrate NaNO_3_ (Merck, 99%); sodium cyanide NaCN (Merck, 96%); potassium thiocyanate (Merck, 99%); thiourea NH_2_CSNH_2_ (Merck, 99%); hydrochloric acid HCl (Panreac, 37%); ethyl alcohol CH_3_CH_2_OH (Merck, 95%); isopropyl alcohol CH_3_CH (OH) CH_3_ (Merck, 98%); deionized water; and granular activated carbon with high strength and low specific surface, obtained from palm kernel by pyrolysis and steam physical activation.

### 3.2. Preparation of Activated Carbons

The functionalization of activated carbon was carried out following the methodology proposed by Muñoz et al. [13], which is a liquid phase impregnation at room temperature. Original activated carbon (2 g) was impregnated with ATG by stirring it with ATG (0.7 g) in 20 mL of isopropyl alcohol for 48 h. Then, the carbon was filtered and washed several times with deionized water. This carbon was filtered again and dried in an oven at 70 °C for 4 h. Finally, it was stored before characterization [14,15]. Unmodified carbon was also washed and dried before the characterization, both impregnated and unmodified carbon were used as adsorbents in the adsorption studies.

### 3.3. Gold Adsorption Studies

#### 3.3.1. Preparation of the Adsorption Solution

The adsorption solution was prepared according to the procedure reported by Yu et al. [22]. To this end, 10 mL of a gold standard solution (1000 mg·L^−1^) were added drop-by-drop to 900 mL of a sodium thiosulfate solution (0.1 mol·L^−1^). Simultaneously, the pH of this solution was kept at 10.00 by the addition of a solution of NaOH (1%). The resulting mixture was transferred to a 1000 mL volumetric flask; thiosulfate solution was added up to the mark of the flask. The solution was then stored in refrigeration at 4 °C, and protected from light [22].

#### 3.3.2. Adsorption Tests

The adsorption tests were carried out as follows: 50 mL of the synthetic solution were stirred with 0.25, 0.5, 1.5, and 2.5 g of carbon in order to attain the desired carbon concentrations (5, 10, 30, and 50 g·L^−1^). These mixtures were then stirred for 24 h. Once stirring was finished, the samples were filtered. The remaining gold content in the solutions was analyzed by atomic absorption spectroscopy in a Perkin Elmer AAnalyst 300 (Perkin Elmer, Shelton, CT, USA) [4]. In order to determine the influence of ATG in the aqueous solution, instead of impregnating it in activated carbon, different ATG quantities were added during the preparation of synthetic solutions to obtain ATG concentrations of 0.001, 0.005, 0.02, and 0.05 mol·L^−1^ [14,15,27].

The recovery rate and capacity of gold adsorption on activated carbon was calculated using Equation (6):

(6) $$R\;=\frac{C_{0}-C_{t}}{C_{0}}\times 100\;\%,$$

where R (%) is the recovery rate of activated carbon for gold, and C_0_ (mg·L^−1^) and C_t_ (mg·L^−1^) are the concentrations of gold in the [Au(S_2_O_3_)_2_]^3−^ solution at time 0 (initial) and time t, respectively.

The loading ability of activated carbon was determined using the Equation (7):

(7) $$Q\;=\frac{R\times C_{0}\times V}{M},$$

where Q (mg × g^−1^) is the capacity of activated carbon, R (%) is the recovery rate of activated carbon for gold, M (g) is the quality of activated carbon used, and V (L) is the volume of the solution.

The adsorption results were analyzed according to the Langmuir model and are presented through Equation (8) [44].

(8) $$\frac{C_{e}}{q_{e}}=\frac{1}{q_{m}}\times C_{e}+\frac{1}{K\times q_{m}}\;$$

where $q_{e}$ (mg × g^−1^) is the capacity of activated carbon, $C_{e}$ (mg·L^−1^) is concentrations of gold, K = ka × kd^−1^, and $q_{m}$ are Langmuir constants. (the adsorption result are presented on Appendix C).

#### 3.3.3. Elution Tests

Elution tests were performed with the impregnated carbon with ATG, using a solution ratio of 50 g·L^−1^, with a stirring speed of 30 rpm for 24 h. For gold elution from the resin, different eluent solutions were evaluated at different concentrations at room temperature: potassium thiocyanate ([KSCN] = 1.0 mol·L^−1^; pH = 12.0; t = 24 h), thiourea ([CH_4_N_2_S] = 0.3 mol·L^−1^; pH = 6.0; t = 24 h), sodium thiosulfate ([Na_2_S_2_O_3_] = 1.0 mol·L^−1^; pH = 11.5; t = 24 h), and sodium cyanide ([NaCN] = 0.2 mol·L^−1^; [NaOH] = 0.25 mol·L^−1^; [CH_3_CH_2_OH] = 30% *V/V*; pH = 12.0; t = 24 h). The carbon charged with precious metals was added to each elution solution and mixed using a magnetic stirrer for 24 h. Once the elution process was finished, filtration was carried out separating the strong solution from the eluted carbon. Afterward, the carbon was washed with deionized water to elute the remaining gold. The strong solution was analyzed by atomic absorption spectrophotometry using a Perkin Elmer AAnalyst 300. The eluted carbon was dried, and the gold retained was evaluated by fire essay. The gold recovery in the elution solutions was determined with Equation (6) as presented in Section 3.3.2 [4].

### 3.4. Characterization of Activated Carbons

Carbons were characterized as follows: specific surface area through the BET method in a Quantachrome Instruments Nova4200e (Quantachrome Instruments, Boynton Beach, FL, USA), functional groups in a PerkinElmer MIR/NIR spectrometer (PerkinElmer, Waltham, MA 02451 USA), morphology and elemental analysis in a scanning electron microscope Tescan Vega LMU Scanning Electron Microscope with X-ray dispersion microanalyzer (SEM-EDS), and zero charge point (PZC), hardness, and proximal analysis [27] through the methods described below.

The PZC was determined by the Mass Titration Method. First, 50 mL of a solution of sodium nitrate (0.1 mol·L^−1^) were added to 100 mL conic flasks. Then, increasing amounts of the adsorbent in the range of 0.5 to 1.6 g were added. The flasks were stirred for 24 h, after which the final pH of the mixture was measured. Thus, a curve of pH versus the amount of absorbent was obtained being the zero charge point located towards the asymptotic part of the curve [45].

The hardness number measures the resistance of carbon to erosion and rupture. These tests were applied according to ASTM D3802-16: Standard test method for ball-pan hardness of activated carbon [46].

The proximal analysis consisted of determining properties, such as moisture (ASTM D2867: Standard test methods for moisture in activated carbon), volatile matter content (ASTM D5832: Standard test method for volatile matter content of activated carbon samples), total ashes content (ASTM D2866: Standard test method for total ash content of activated carbon) and the fixed carbon content. The value of fixed carbon content was calculated as the subtraction of the initial weight of the carbon minus the moisture, volatile, and ash contents [47,48,49].

### 3.5. Characterization of Gold Thiosulphate and Gold Thiolate Synthetic Solutions

To study the electrochemistry characteristics of thiosulphate and gold thiolate synthetic solutions, a CH-Instruments Potentiostat (Tennison Hill Drive Austin, TX, USA), Model 700D, was used coupled to a conventional 15 mL three electrode reaction cell. The working electrode was a gold electrode (surface 4.522 mm^2^), whose potential was controlled against the saturated calomel reference electrode (SCE). A graphite bar (r = 2 mm and L = 5 cm) served as a counter electrode. All experiments were carried out at room temperature (15 °C). Cyclic voltammograms were recorded with a scan rate of 100 mV/s, between −1.0 to 1.2 mV as the potential range [32].

The gold complex in solution was identified using ultraviolet–visible spectroscopy using a Lambda Bio+ UV–vis spectrophotometer (PerkinElmer, Waltham, MA 02451 USA). In these experiments, the spectra were recorded using a standard quartz cuvette with a particular electrolyte, and by performing a fast scan over the wavelength of 190–900 nm [32,37].

## 4. Conclusions

In this paper, the results of the adsorption process of a gold thiosulfate complex on thiol-modified activated carbon were presented. The adsorption of ATG in activated carbon allowed for a recovery of 90% of the gold in solution using 50 g·L^−1^ of carbon.

The volatile matter content increased from 10 to 14%, and the point of zero charge decreased from 9.0 to 3.2 as a result of the impregnation process of activated carbon. The characterization by FTIR and SEM-EDS of the carbon impregnated with ATG also evidenced the insertion of sulfur groups on the structure of activated carbon.

The presence of thioglycolate ions in carbon or in the synthetic solutions improved gold recovery with activated carbon. As the concentration of thioglycolic acid increased, gold recovery increased, reaching a recovery of 89.9% at 0.05 mol L^−1^ of ATG, 50 g·L^−1^ of carbon, 10 mg·L^−1^ of carbon, and 0.1 mol·L^−1^ thiosulfate.

Elution tests showed a strong affinity of the gold thioglycolate complex for activated carbon, since the four eluents tested did not exceed 50% of gold recovery.

Based on UV-VIS spectrometry and cyclic voltammetry, the existence of a new gold thiolate complex was demonstrated. This complex was responsible of the gold recovery with activated carbon and was formed by the interaction between the thioglycolate ion and the gold thiosulfate complex. Thiol groups could break the disulfide bond of the aurothiosulfate complex and form a new gold complex.

## Figures and Tables

**Table 1:** Gold recovery from ATG-impregnated carbon, 50 g·L^−1^ of carbon, T = 90 °C, t = 24 h.

Eluent Solutions	Gold Recovery (%)
[KSCN] = 1.0 mol·L^−1^	25.8
[CH_4_N_2_S] = 0.3 mol·L^−1^	31.8
[Na_2_S_2_O_3_] = 1.0 mol·L^−1^	26.1
[NaCN] = 0.2 mol·L^−1^; [NaOH] = 0.25 mol·L^−1^; [CH_3_CH_2_OH]= 30% *V/V*	44.9

**Table 2:** Characterization of ATG-impregnated carbon and unmodified activated carbon.

Description	Unmodified Carbon	ATG-Impregnated Carbon
Diameter (mm)	4.8	4.8
Volatile matter (%)	10.0	13.9
Fixed carbon (%)	68.9	68.0
Specific surface area (m^2^ × g^−1^)	274.0	-
Hardness (%)	90.3	89.1
Acidity (mmol × g^−1^)	0.05	-
Point of zero charge (PZC)	9.80	3.20

**Figure A4:**
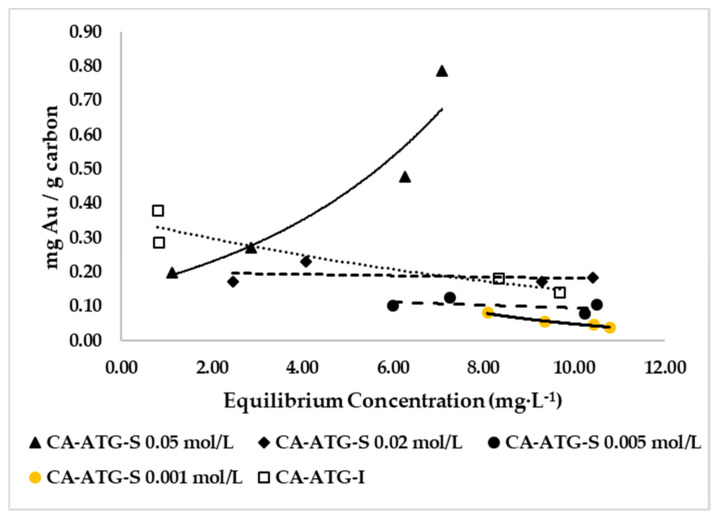
Gold adsorption isotherms of activated carbon stirred with ATG (0.001, 0.005, 0.02, and 0.05 mol·L^−1^) and modified carbon impregnated with ATG. (**S** represents activated carbon stirred with ATG in aqueous solution and **I** represent activated carbon impregnated with ATG).

## Appendix B

### Elemental Analysis of the thiol-impregnated Activated Carbon by Scanning Electron Microscope (SEM-EDS)

**Table A1:** Elemental analysis of thiol-impregnated activated carbon.

Activated Carbon	C	O	Si	S	K	Fe	Mg
(%)	93.36	2.37	2.08	1.96	1.55	0.44	0.42

The gold balance of a sample of carbon obtained from adsorption tests is also presented in Table A2, it evidences a gold recovery of 89% in the activated carbon.

**Table A2:** Gold balance of a sample of activated carbon obtained from adsorption tests.

Description	Au (ppm)	Dilution	Recovered Volume (mL)	Au (mg)	Au (%)
Initial Solution	11.78	1	50	0.589	NA
Strong Solution	1.34	1	43	0.057	9.5%
Wash solution	0.23	1	45	0.010	1.7%
Carbon	NA	NA	NA	0.530	88.7%
Re-calculated feed				0.597	100.0%

## Appendix C

The adsorption results were analyzed according to Langmuir models, the adsorption constants (K) determined through the Equation (A1) are presented in Table A3. The results show a direct effect of the ATG concentration on the value of the adsorption constant. This value increased from 0.25 to 3.52 when the ATG concentration increased. In addition, the adsorption of activated carbon with ATG (0.05 mol·L^−1^) stirred in aqueous solution is better than the adsorption of carbon impregnated with ATG. This improvement in the adsorption is linked with the equivalent concentration of ATG that is impregnated in the carbon, which is approximately 0.02 mol·L^−1^. Gold adsorption isotherms of activated carbons (stirred with ATG and impregnated with ATG) are displayed in Figure A3.

A1 $$\frac{C_{e}}{q_{e}}=\frac{1}{q_{m}}\times C_{e}+\frac{1}{K\times q_{m}}\;$$

**Table A3:** Langmuir constants and correlation factor for adsorption isotherms of activated carbon stirred with ATG (0.001, 0.005, 0.02, and 0.05 mol·L^−1^) and modified carbon impregnated with ATG.

Description	r2	qm	K(ka/kd)
CA-ATG-S 0.001 mol/L	0.7938	0.0016	0.2458
CA-ATG-S 0.005 mol/L	0.8064	0.0002	0.1576
CA-ATG-S 0.02 mol/L	0.9396	0.0063	1.1049
CA-ATG-S 0.05 mol/L	0.7887	0.0103	3.5244
CA-ATG-I	0.8429	0.0111	1.7839

## Sample Availability

Samples of the compounds are available from the authors.

## References

[1] de la Torre E., Gámez S., Pazmiño E., Francesco V., Ionela B. (2018). Improvements to the cyanidation process for precious metal recovery from WPCBs. Waste Electrical and Electronic Equipment Recycling.

[2] Xu B., Kong W., Li Q., Yang Y., Jiang T., Liu X. (2017). A Review of Thiosulfate Leaching of Gold: Focus on Thiosulfate Consumption and Gold Recovery from Pregnant Solution. Metal.

[3] O’Malley G. (2002). Recovery of Gold from Thiosulfate Solutions and Pulps with Ion-Exchange Resins. Ph.D. Thesis.

[4] Gámez S., Garcés K., De La Torre E., Guevara A. (2019). Precious metals recovery from waste printed circuit boards using thiosulfate leaching and ion exchange resin. Hydrometallurgy.

[5] Liu X., Xu B., Min X., Li Q., Yang Y., Jiang T., He Y., Zhang X. (2017). Effect of Pyrite on Thiosulfate Leaching of Gold and the Role of Ammonium Alcohol Polyvinyl Phosphate (AAPP). Metals.

[6] Ha V.H., Lee J.-C., Huynh T.H., Jeong J., Pandey B. (2014). Optimizing the thiosulfate leaching of gold from printed circuit boards of discarded mobile phone. Hydrometallurgy.

[7] Dong Z., Jiang T., Xu B., Yang Y., Li Q. (2019). An eco-friendly and efficient process of low potential thiosulfate leaching-resin adsorption recovery for extracting gold from a roasted gold concentrate. J. Clean. Prod..

[8] Sitando O., Senanayake G., Dai X., Breuer P.L. (2019). The adsorption of gold(I) on minerals and activated carbon (preg-robbing) in non-ammoniacal thiosulfate solutions - effect of calcium thiosulfate, silver(I), copper(I) and polythionate ions. Hydrometallurgy.

[9] Aylmore M., Muir D. (2001). Thiosulfate leaching of gold—A review. Miner. Eng..

[10] Navarro P., Vargas C., Alonso M., Alguacil F.J. (2007). Towards a more environmentally friendly process for gold: Models on gold adsorption onto activated carbon from ammoniacal thiosulfate solutions. Desalination.

[11] Navarro P., Vargas C., Alonso M., Alguacil F.J. (2006). The adsorption of gold on activated carbon from thiosulfate-ammoniacal solutions. Gold Bull..

[12] Dong Z., Jiang T., Xu B., Yang Y., Li Q. (2017). Recovery of Gold from Pregnant Thiosulfate Solutions by the Resin Adsorption Technique. Metals.

[13] Muñoz M., Aller A.-J., Littlejohn D. (2014). The bonding of heavy metals on nitric acid-etched coal fly ashes functionalized with 2-mercaptoethanol or thioglycolic acid. Mater. Chem. Phys..

[14] Chen Y., Zi F., Hu X., Yang P., Ma Y., Cheng H., Wang Q., Qin X., Liu Y., Chen S. (2020). The use of new modified activated carbon in thiosulfate solution: A green gold recovery technology. Sep. Purif. Technol..

[15] Chen Y., Zi F., Hu X., Yu H., Nie Y., Yang P., Cheng H., Wang Q., Qin X., Chen S. (2019). Grafting of organic sulfur-containing functional groups on activated carbon for gold(I) adsorption from thiosulfate solution. Hydrometallurgy.

[16] Zhou Z., Anderson C.M., Butler S.K., Thompson S.K., Whitty K.J., Shen T.-C., Stowers K.J. (2017). Stability and efficiency of CO 2 capture using linear amine polymer modified carbon nanotubes. J. Mater. Chem. A.

[17] Fotoohi B., Mercier L. (2015). Some insights into the chemistry of gold adsorption by thiol and amine functionalized mesoporous silica in simulated thiosulfate system. Hydrometallurgy.

[18] Ewecharoen A., Thiravetyan P., Wendel E., Bertagnolli H. (2009). Nickel adsorption by sodium polyacrylate-grafted activated carbon. J. Hazard. Mater..

[19] Jang M., Cannon F.S., Parette R.B., Yoon S.-J., Chen W. (2009). Combined hydrous ferric oxide and quaternary ammonium surfactant tailoring of granular activated carbon for concurrent arsenate and perchlorate removal. Water Res..

[20] Shafeeyan M.S., Daud W.M.A.W., Houshmand A., Shamiri A. (2010). A review on surface modification of activated carbon for carbon dioxide adsorption. J. Anal. Appl. Pyrolysis.

[21] Bandosz T., Ania C.O. (2006). Chapter 4 Surface chemistry of activated carbons and its characterization. Interface Sci. Technol..

[22] Yu H., Zi F., Hu X., Nie Y., Chen Y., Cheng H. (2017). Adsorption of gold from thiosulfate solutions with chemically modified activated carbon. Adsorpt. Sci. Technol..

[23] Melashvili M., Fleming C., Dymov I., Matthews D., Dreisinger D. (2015). Equation for thiosulphate yield during pyrite oxidation. Miner. Eng..

[24] Melashvili M., Fleming C., Dymov I., Matthews D., Dreisinger D. (2016). Dissolution of gold during pyrite oxidation reaction. Miner. Eng..

[25] Grosse A.C., Dicinoski G.W., Shaw M.J., Haddad P.R. (2003). Leaching and recovery of gold using ammoniacal thiosulfate leach liquors (a review). Hydrometallurgy.

[26] Staunton W. (2016). Carbon-in-Pulp.

[27] Toledo R.B.C., Aragón-Tobar C.F., Gámez S., De La Torre E. (2020). Reactivation Process of Activated Carbons: Effect on the Mechanical and Adsorptive Properties. Molecules.

[28] Houshmand A., Shafeeyan M.S., Arami-Niya A., Daud W.M.A.W. (2013). Anchoring a halogenated amine on the surface of a microporous activated carbon for carbon dioxide capture. J. Taiwan Inst. Chem. Eng..

[29] Mohammadnejad S., Provis J.L., Van Deventer J.S.J. (2015). Computational modelling of gold complexes using density functional theory. Comput. Theor. Chem..

[30] Abechi S.E., Gimba C.E., Uzairu A., Dallatu Y.A. (2013). Preparation and Characterization of Activated Carbon from Palm Kernel Shell by Chemical Activation. Res. J. Chem. Sci..

[31] Kasper A.C., Carrillo-Abad J., García-Gabaldón M., Veit H.M., Pérez-Herranz V. (2015). Determination of the potential gold electrowinning from an ammoniacal thiosulphate solution applied to recycling of printed circuit board scraps. Waste Manag. Res..

[32] Dimitrijevic S., Rajčić-Vujasinović M., Alagić S.Č., Grekulović V., Trujic V. (2013). Formulation and characterization of electrolyte for decorative gold plating based on mercaptotriazole. Electrochimica Acta.

[33] Sitando O., Senanayake G., Dai X., Nikoloski A., Breuer P.L. (2018). A review of factors affecting gold leaching in non-ammoniacal thiosulfate solutions including degradation and in-situ generation of thiosulfate. Hydrometallurgy.

[34] Osorio H.M., Cea P., Ballesteros L.M., Gascón I., Marqués-González S., Low P.J., Nichols R.J., Perez F., Martín S. (2014). Preparation of nascent molecular electronic devices from gold nanoparticles and terminal alkyne functionalised monolayer films. J. Mater. Chem. C.

[35] Laurentius L.B., Stoyanov S.R., Gusarov S., Kovalenko A., Du R., Lopinski G.P., McDermott M. (2011). Diazonium-Derived Aryl Films on Gold Nanoparticles: Evidence for a Carbon–Gold Covalent Bond. ACS Nano.

[36] Yang Y.-B., Zhang X., Xu B., Li Q., Jiang T., Wang Y. (2015). Effect of arsenopyrite on thiosulfate leaching of gold. Trans. Nonferrous Met. Soc. China.

[37] Jia W.-G., Dai Y.-C., Zhang H.-N., Lu X., Sheng E.-H. (2015). Synthesis and characterization of gold complexes with pyridine-based SNS ligands and as homogeneous catalysts for reduction of 4-nitrophenol. RSC Adv..

[38] Zawadzki H.J. (2003). Synthesis and spectral studies of gold(III) complexes with guanidine derivatives. Transit. Met. Chem..

[39] Al-Maythalony B.A., Wazeer M.I., Isab A.A. (2010). 1H, 13C NMR and UV spectroscopy studies of gold(III)-tetracyanide complex with l-cysteine, glutathione, captopril, l-methionine and dl-seleno-methionine in aqueous solution. Inorg. Chim. Acta.

[40] Wang J., Li X., He Y., Song P., Wang R. (2018). Preparation of Keratin-Glycine Metal Complexes and Their Scavenging Activity for Superoxide Anion Radicals. Int. J. Polym. Sci..

[41] De Guzman R., Tsuda S.M., Ton M.-T.N., Zhang X., Esker A.R., Van Dyke M.E. (2015). Binding Interactions of Keratin-Based Hair Fiber Extract to Gold, Keratin, and BMP-2. PLoS ONE.

[42] Parker A.J., Kharasch N. (1959). The Scission of the Sulfur-Sulfur Bond. Chem. Rev..

[43] Bachrach S.M., Woody J.T., Mulhearn D.C. (2002). Effect of Ring Strain on the Thiolate−Disulfide Exchange. A Computational Study. J. Org. Chem..

[44] Kumar K.V. (2007). Optimum sorption isotherm by linear and non-linear methods for malachite green onto lemon peel. Dye. Pigment..

[45] Mahmood T., Saddique M.T., Naeem A., Westerhoff P., Mustafa S., Alum A. (2011). Comparison of Different Methods for the Point of Zero Charge Determination of NiO. Ind. Eng. Chem. Res..

[46] ASTM International (2016). D3802-16 Standard Test Method for Ball-Pan Hardness of Activated Carbon.

[47] ASTM International (2014). D5832-98(2014) Standard Test Method for Volatile Matter Content of Activated Carbon Samples.

[48] ASTM International (2018). D2866-11(2018) Standard Test Method for Total Ash Content of Activated Carbon.

[49] ASTM International (2017). D2867-17(2017) Standard Test Methods for Moisture in Activated Carbon.

